# Neonatal surveillance for congenital Zika infection during the 2016 microcephaly outbreak in Salvador, Brazil: Zika virus detection in asymptomatic newborns

**DOI:** 10.1002/ijgo.13042

**Published:** 2020-01-23

**Authors:** João V. Oliveira, Tereza C.X. Carvalho, Marta Giovanetti, Jaqueline G. de Jesus, Cleiton S. Santos, Lorena B. Pessoa, Cláudio F.Q. Magalhães Filho, Jéssica G.S. Lima, Daniel A.X. Carvalho, Eduardo M. Figueiredo, Ana Carolina Biron, Daiana C. dos Santos, Paloma Viana, Alan O. Duarte, Rosana Pessoa, Gloryane B. Souza, Juan I. Calcagno, Fernanda W.M. Lima, Luiz C.J. Alcantara, Isadora C. de Siqueira

**Affiliations:** ^1^ Gonçalo Moniz Institute Oswaldo Cruz Foundation Ministry of Health Salvador BA Brazil; ^2^ José Maria de Magalhães Netto Maternity Hospital Salvador BA Brazil; ^3^ Flavivirus Laboratory Oswaldo Cruz Institute Fiocruz, Rio de Janeiro RJ Brazil; ^4^ Cell and Molecular Genetics Laboratory ICB Federal University of Minas Gerais, Belo Horizonte MG Brazil; ^5^ Immunology of Infectious Diseases Laboratory Faculty of Pharmacy Federal University of Bahia Salvador BA Brazil

**Keywords:** Brazil, Congenital infection, Microcephaly, Neonatal surveillance, Zika virus

## Abstract

**Objective:**

To identify newborns with congenital Zika infection (CZI) at a maternity hospital in Salvador, Brazil, during the 2016 microcephaly outbreak.

**Methods:**

A prospective study enrolled microcephalic and normocephalic newborns with suspected CZI between January and December 2016. Serology (immunoglobulins IgM and IgG) and quantitative reverse transcriptase polymerase chain reaction (RT‐qPCR) for the Zika virus were performed. Demographic and clinical characteristics of newborns with and without microcephaly were compared.

**Results:**

Of the 151 newborns enrolled, 32 (21.2%) were classified as microcephalic. The majority of these cases were born between January and May 2016. IgM and IgG Zika virus antibodies were detected in 5 (23.8%) and 17 (80.9%) microcephalic newborn blood samples, respectively. Six (24%) microcephalic newborns tested positive for Zika virus by RT‐qPCR in urine or placenta samples. Thirteen (11.8%) normocephalic newborns also tested positive for Zika virus by PCR in urine, plasma, or placenta samples, while IgM antibodies against Zika were detected in 4 (4.2%) others.

**Conclusions:**

Identification of 17 normocephalic CZI cases, confirmed by IgM serology or RT‐qPCR for Zika virus, provides evidence that CZI can present asymptomatically at birth. This finding highlights the need for prenatal and neonatal screening for Zika virus in endemic regions.

## Abstract 2

Neonatal surveillance for congenital Zika infection in Brazil and the identification of Zika virus in microcephalic and normocephalic newborns.

## 1 INTRODUCTION

In May 2015, the transmission of Zika virus was confirmed in Brazil.^1^ During the Zika outbreak in Salvador, Brazil, 14 835 suspected cases were reported.^2^ In total, the Brazilian Ministry of Health estimated that 440 000–1.3 million individuals were infected by Zika virus in 2015.^3^ Infection was initially considered benign and self‐limiting.^4^, ^5^

In late 2015, an unexpected outbreak of newborns with microcephaly occurred in major cities in northeastern Brazil and a state of public health emergency was declared in the country. Between October and December 2015, 2975 cases of microcephalic newborns were notified in Brazil, mostly in the states of Pernambuco (1153 cases), Paraíba (476 cases), and Bahia (271 cases),^6^ with 229 cases notified in the city of Salvador—the capital of the state of Bahia.^7^

In response to the microcephaly outbreak in northeastern Brazil, the Pan American Health Organization (PAHO) and the World Health Organization (WHO) released an epidemiological alert,^8^ and a rapid risk assessment by the European Centre for Disease Prevention and Control (ECDC) highlighted a possible link between increased rates of congenital microcephaly in Brazil and the Zika virus epidemic.^9^

The association between microcephaly and congenital Zika infection (CZI) was established by the identification of Zika virus in the amniotic fluid of a pregnant woman in Brazil^10^ and in the brain tissue of a fetus whose mother was infected by Zika virus.^11^ By the end of 2017, a total of 15 298 notified cases of CZI were reported in Brazil, of which 3071 were confirmed.^12^

The aim of the present study was to identify and characterize cases of CZI at a maternity hospital in Salvador, Bahia, during the 2016 microcephaly outbreak.

## 2 MATERIALS AND METHODS

This prospective study was conducted at the Jose Maria Magalhães Netto public maternity hospital located in Salvador, Bahia, Brazil, between January 18 and December 16, 2016.

In accordance with the protocol established by the Brazilian Ministry of Health in November 2015,^13^ the head circumference of newborns was measured at this maternity hospital by health professionals and cases of microcephaly were identified following specific criteria. Newborns who fulfilled these criteria were reported to local health authorities, and enrollment consent was requested from legal guardians to participate in this study. In addition, enrollment was also requested for newborns without microcephaly whose mothers reported episodes of exanthematous skin rash during pregnancy. All newborns were enrolled within the first 24 hours after birth.

Sociodemographic data and clinical information from mothers were collected through interviews, while clinical data on newborns and birth conditions were obtained by reviewing medical records. Data entry and data management were performed using REDCap version 6.18.1 (Vanderbilt University, Nashville, TN, USA). Biological samples, including placental tissue, blood, umbilical cord blood, and newborn urine were collected by nurses in the maternity ward and the laboratory team at the hospital. Serological and molecular tests were done at the Gonçalo Moniz Institute, Oswaldo Cruz Foundation, Salvador (IGM‐FIOCRUZ) and at the Infectious Diseases Immunology Laboratory of the Federal University of Bahia, Salvador.

During the study period, the Brazilian Ministry of Health changed the protocol for the specific criteria used to identify cases of microcephaly. From January to March 12, 2016, the cutoff for microcephaly notification for term newborns was a head circumference of less than or equal to 32 cm for both sexes; and less than or equal to the third percentile on the Fenton growth chart by gestational age and sex for preterm newborns. Between March 13 and December 2016, new criteria were employed to determine microcephaly cases: head circumference less than or equal to two standard deviations below the average (WHO standard) for term newborns; and less than or equal to two standard deviations below the average (INTERGROWTH‐21st) by gestational age and sex for preterm newborns.^14^

For data analysis purposes, all enrolled newborns were reclassified to define microcephaly according to the International Fetal and Newborn Growth Consortium for the 21st Century (INTERGROWTH‐21st) charts, taking into account the newborn's gender, gestational age, and head circumference at birth.^15^ Microcephaly was defined as head circumference measuring less than two standard deviations below the average, while severe microcephaly was considered if head circumference measurements were less than three standard deviations below the average. Newborns were considered normocephalic if head circumference measurements were within two standard deviations.

Newborns were classified as small, appropriate, or large for their gestational age based on gender‐specific birth weight for their gestational age using WHO reference curves.^16^ Newborns considered small‐for‐gestational‐age (SGA) were defined as having a weight below the 10th percentile of the corresponding standard reference curve.

Transfontanellar ultrasound was performed as a standard procedure by the medical staff at the maternity hospital during admission. Information regarding these procedures was obtained from patient medical records.

For serological analysis, detection of specific anti‐Zika virus immunoglobulin G (IgG) antibodies using indirect enzyme‐linked immunosorbent assays (ELISA) (EUROIMMUN; Lüberg, Germany) was performed in accordance with the manufacturer protocol. In addition, an immunoglobulin M (IgM) antibody capture ELISA (MAC‐ELISA), provided by the Arbovirus Reference Collection (ARC) division of the Centers for Disease Control and Prevention (CDC), was used in accordance with the established CDC protocol.^17^

*Cytomegalovirus* IgM (Dia.Pro SRL; Milan, Italy) and *Toxoplasma* IgM (Dia.Pro) were also assayed by ELISA in blood samples of newborns whose mothers tested positive (IgM serology). Serum Venereal Disease Research Laboratory (VDRL) test results and HIV status were obtained from patient medical records. *Cytomegalovirus* IgG and *Toxoplasma* IgG results from mothers were obtained from medical records.

For Zika virus diagnosis, viral RNA was extracted from clinical samples using the QIAmp viral RNA mini kit (Qiagen; Hilden, Germany) and quantitative reverse transcriptase polymerase chain reaction (RT‐qPCR) was performed for Zika virus, as previously described.^18^

Data analysis was performed using SPSS version 21 (IBM, Armonk, NY, USA) software. Comparison of demographic and clinical characteristics of newborns with and without microcephaly was done using either the χ^2^ or Fisher exact test for categorical variables or the Mann‐Whitney *U* test or Kruskal‐Wallis test for continuous variables. *P*<0.05 was considered statistically significant.

The study was approved by the Institutional Review Board of the Gonçalo Muniz Institute, Oswaldo Cruz Foundation (IGM‐FIOCRUZ, protocol no. 1.935.854/2016) and by the Ethical Review Committee of the Pan‐American Health Organization (PAHOERC 2017‐05‐0044). The legal guardians of all newborns provided written informed consent.

## 3 RESULTS

Between January and December 2016, 172 newborns were identified as eligible by health professionals at the maternity hospital in Salvador, Brazil. Of these, 21 of the infants’ guardians declined to participate in the study, resulting in 151 newborns subsequently enrolled: 32 (21.2%) classified as microcephalic and 119 (78.8%) as normocephalic.

**Figure 1:**
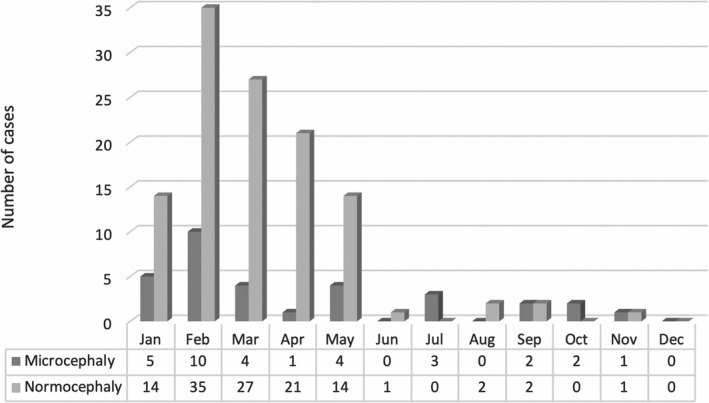
Temporal distribution of enrolled cases by month of birth (January to December 2016).

The temporal distribution of cases by month of birth is shown in Figure 1. A cluster of cases born between January and May was observed. Maternal sociodemographic data are presented in Table 1. No differences in the mothers’ age, race, or educational level were observed between microcephalic and normocephalic newborns (*P*>0.05).

**Table 1:** Sociodemographic and clinical data for 151 newborns from Salvador, Bahia‐Brazil, 2016.^a^

Characteristics	Microcephalic newborns (n=32)	Normocephalic newborns (n=119)	*P* value
Mothers’ age, y	23 (19.5–30.5)	23 (19.5–29.50)	0.98
Mothers’ declared skin color			
Black	16 (50.0)	63 (52.9)	0.14
Brown	6 (18.8)	37 (31.0)	
White	2 (6.3)	3 (2.5)	
Other	2 (6.3)	1 (0.8)	
Unknown	6 (18.8)	15 (12.6)	
Mothers’ education			
Grade school	14 (43.8)	51 (42.8)	0.49
Secondary school	15 (46.9)	56 (47.0)	
College	3 (9.4)	6 (5.0)	
Unknown	0	6 (5.0)	
Type of delivery			
Vaginal	26 (81.3)	77 (64.7)	0.08
Cesarean	6 (18.8)	42 (35.3)	
Newborns’ gender			
Female	19 (59.4)	53 (44.5)	0.26
Male	13 (40.6)	66 (55.4)	
Gestational age at birth, wk	39.0 (38.0–40.0)	39 (38.0–40.0)	0.30
Head circumference, cm	31.0 (31.0–31.5)	34.0 (33.0–35.0)	<0.001
Length at birth, cm	46.0 (42.75–46.63)	47.75 (46.0–49.0)	<0.001
Weight at birth, kg	2.64 (2.30–2.92)	3.09 (2.83–3.40)	<0.001
Size for gestational age			
Small for gestational age	18 (56.3)	14 (11.8)	<0.001
Appropriate for gestational age	14 (43.8)	101 (84.9)	
Large for gestational age	0 (0.0)	4 (3.4)	

a Values are given as number (percentage) or median (interquartile range), unless otherwise indicated.

The median gestational age at the time of delivery was 39 weeks (IQR, 38–40) and most (n=103, 68.2%) deliveries were vaginal. Seventy‐nine (52.3%) newborns were male. A total of 115 (76.1%) newborns were normal birth weight for their gestational age, while 32 (21.2%) were classified as SGA (Table 1.)

Based on INTERGROWTH‐21st criteria, 32 (21.2%) newborns were classified as microcephalic, and 5 (15.6%) of these were classified as having severe microcephaly; 119 (78.8%) were considered normocephalic. Median head circumference at birth was 34.0 cm (IQR, 32.0–35.0) and 31.0 cm (IQR, 31.0–31.5) for normocephalic and microcephalic newborns, respectively.

No differences in gestational age at birth were seen between the microcephalic and normocephalic newborns (*P*=0.30). However, significantly higher proportions of microcephalic newborns had low birth weight (*P*<0.001) and were SGA (*P*<0.001) compared with normocephalic newborns (Table 1).

Eight (25.0%) microcephalic newborns and 9 (7.6%) normocephalic newborns presented abnormal Apgar scores within the first minute of life. Transfontanellar ultrasound was performed in 123 (81.5%) of the enrolled newborns. Of 28 microcephalic newborns who underwent ultrasound, 7 (25.0%) had abnormal results, with the presence of calcifications observed in all seven cases, ventriculomegaly in 4 (14.3%) and the absence of corpus callosum in one (3.5%) case.

Arthrogryposis was observed in 2 (6.3%) microcephalic newborns: detected in the upper and lower limbs of one and in only the lower extremities of the other. Both newborns presented bilateral clubfoot and bilateral hip dislocation.

Twenty‐five samples of plasma, urine, or placental tissue were available for molecular diagnosis from microcephalic newborns. Six (24.0%) tested positively for Zika virus by RT‐qPCR in two samples of urine and four of placental tissue, while no positivity was detected in any of the plasma samples. Serological testing for Zika virus was performed in 21 plasma samples from microcephalic newborns, with IgM positivity observed in 5 (23.8%) versus IgG positivity in 17 (80.9%). In all, 19 (76.0%) microcephalic newborns presented some evidence of Zika virus infection, and 6 (24.0%) tested negative for Zika virus under all procedures. The results of IgG and IgM Zika‐specific antibodies and RT‐qPCR results for microcephalic newborns are presented in Table 2. Detailed individual test results are available as supporting information Table S1.

**Table 2:** Zika virus serology and RT‐qPCR positivity in newborns from Salvador, Brazil, 2016.^a^

Zika virus serology	Microcephalic newborns	Normocephalic newborns	All newborns	*P* value
Zika RT‐qPCR positive	6/25 (24.0)	13/110 (11.8)	19/135 (14.1)	0.24
Anti‐Zika IgM positive	5/21 (23.8)	4/95 (4.2)	9/116 (7.7)	0.03
Anti‐Zika IgG positive	17/21 (80.9)	71/95 (74.7)	88/116 (75.9)	0.56

Abbreviations: Ig, immunoglobulin; RT‐qPCR, quantitative reverse transcription polymerase chain reaction.

a Values are given as number (percentage) unless otherwise indicated.

For normocephalic newborns there were 110 samples of plasma, 87 samples of urine, and 100 samples of placental tissue available for molecular diagnosis. Thirteen (11.8%) normocephalic newborns tested positive for Zika virus by RT‐qPCR in seven samples of urine, two of placental tissue, and six of plasma. Serological testing for Zika virus was performed in 95 samples, with IgM positivity observed in 4 (4.2%) versus IgG positivity in 71 (74.7%). A total of 76 (69.1%) normocephalic newborns presented some evidence of Zika virus infection, with 17 confirmed with Zika virus by IgM serology or RT‐qPCR. The results of IgG and IgM Zika‐specific antibodies and RT‐qPCR results for normocephalic newborns are presented in Table 2. Detailed individual test results are available as supporting information Table S2.

All mothers and newborns were tested for syphilis and HIV at hospital admission. Five (3.3%) women had positive VDRL and all were HIV negative. None of the enrolled newborns presented any serological evidence of syphilis or HIV infection. *Toxoplasma* and *Cytomegalovirus* results were available for 123 (81.5%) women. While the majority had previous exposure to *Cytomegalovirus* (n=115, 93.4%) or *Toxoplasma* (n=58, 47.1%), only one presented anti‐*toxoplasma* IgM positivity, and four were anti‐*Cytomegalovirus* IgM‐positive on serology. By contrast, all of their newborns were IgM seronegative for *Cytomegalovirus* and *Toxoplasma*, and none presented any clinical signs of congenital infection.

Twelve (7.9%) newborns were admitted to a neonatal intensive care unit, and 3 (2.0%) died. One of the deaths was attributed to severe microcephaly, and two others to premature birth (both normocephalic newborns).

## 4 DISCUSSION

The present year‐long hospital study was conducted in response to an initial surge in microcephaly cases in October 2015 in Salvador, Brazil. Microcephalic newborns with a clinical suspicion of CZI were enrolled, in addition to normocephalic cases in which the mothers reported the presence of a skin rash (a possible sign of Zika virus infection) at some point during pregnancy.

Elevated cases of microcephalic and normocephalic newborns were observed to cluster together during the first 5 months of 2016, just months after the 2015 Zika virus outbreak in Salvador. This epidemic link was reported previously^7^ and reinforces the role of Zika virus infection with respect to CZI.

Difficulties in confirming the diagnosis of Zika virus in the microcephalic cases have been previously reported.^19^ The rate of anti‐Zika IgM or Zika virus RT‐qPCR positivity observed herein in microcephalic newborns was similar to rates found in a previous study.^20^ However, other authors found higher Zika virus IgM positivity in microcephalic cases than in the present study, mostly in cerebrospinal fluid (CSF) samples^21^; however, CSF samples were not available for analysis in the present study. Regardless, the detection of Zika‐specific IgM by MAC‐ELISA in neonates seems to be an adequate method for CZI diagnosis when CSF sampling is not feasible.^21^

Although microcephalic newborns had a higher rate of anti‐Zika IgM than normocephalic newborns (*P*=0.03), we found a similar rate of Zika virus RT‐qPCR positivity in both groups. Most of the Zika virus RNA identified herein were in urine samples. While Zika virus RT‐qPCR is considered a valuable option for viral RNA identification in samples of urine during acute Zika virus infection, due to the short period of viremia,^22^ the performance of this specimen type has not been previously evaluated in the context of CZI diagnosis. The low rate of Zika virus RNA identified in plasma herein could be related to the lengthy period between the acute phase of Zika virus infection and delivery. Nonetheless, prolonged shedding of viral RNA has been reported, with RT‐PCR positivity detected in blood and also placenta samples.^23^

In the 32 microcephalic cases investigated, severe cases with head circumference below three standard deviations using INTERGROWTH‐21st criteria were observed in 5 (15.6%) newborns, with 1 (3.1%) evolving to death. In addition, arthrogryposis, one of the previously described clinical manifestations of CZI,^24^ was found in 2 (6.2%) newborns. Furthermore, many of the microcephalic newborns herein were small for their gestational age, which is consistent with findings from a previous study that identified an elevated prevalence of SGA in CZI newborns.^20^ However, whether SGA is due to the pathological morbidity of CZI remains an open question.

Vigilant monitoring is necessary in cases of normocephalic CZI, as late‐onset microcephaly has been reported in infants with CZI,^25^ in addition to neurodevelopmental delays in infants with intrauterine exposure to Zika virus.^26^

The inclusion of normocephalic cases in the present study allowed for the additional identification of 17 normocephalic cases of CZI confirmed by IgM serology or RT‐qPCR for Zika virus. Our findings reinforce the notion that CZI holds the potential to present asymptomatically at birth and highlight the necessity to implement prenatal and neonatal Zika virus screening in endemic regions as a standardized protocol.

## AUTHOR CONTRIBUTIONS

JVO, FWML, and ICS contributed to the study design, writing of the manuscript, and data analysis. JVO, TCXC, LBP, CMF, JGSL, DAC, EMF, ACB, RP, GBS, and JIC contributed with the enrollment of participants, reviewed medical records, and collected samples and data. LCJA, MG, JJG, DCS, PV, AOD, CSS, FWML, and ICS contributed with laboratory analysis.

## CONFLICTS OF INTEREST

The authors have no conflicts of interest.

## Supporting information


**Table S1.** Laboratory and clinical characteristics of 25 newborns with microcephaly and available samples, Salvador, 2016.
**Table S2.** Laboratory and clinical characteristics of 17 normocephalic newborns with congenital Zika infection (IgM anti‐Zika virus or Zika virus RT‐qPCR positive).
