# Supplementary material for: Neonatal surveillance for congenital Zika infection during the 2016 microcephaly outbreak in Salvador, Brazil: Zika virus detection in asymptomatic newborns
Source: Int J Gynaecol Obstet. 2020 Jan 23;148(Suppl 2):9–14. doi: 10.1002/ijgo.13042 (PMC7064952; doi:10.1002/ijgo.13042)
Supplement: Supplementary file 1 — Table S1. Laboratory and clinical characteristics of 25 newborns with microcephaly and available samples, Salvador, 2016. Table S2. Laboratory and clinical characteristics of 17 normocephalic newborns with congenital Zika infection (IgM anti‐Zika virus or Zika virus RT‐qPCR positive). [file IJGO-148-9-s001.docx]

**Supporting information** **S1.** Laboratory and clinical characteristics of 25 newborns with microcephaly and available samples, Salvador, 2016.

| Subject ID | IgG anti-Zika virus | IgM anti-Zika virus | Zika RT-qPCR | Size for gestational age | Head circumference, cm | Intergrowth classification |
| --- | --- | --- | --- | --- | --- | --- |
| 01 | **Positive** | **Positive** | Negative | AGA | 27.0 | Severe microcephaly |
| 02 | **Positive** | Negative | Negative | SGA | 30.0 | Microcephaly |
| 03 | **Positive** | Negative | Negative | AGA | 29.0 | Severe microcephaly |
| 04 | Negative | Negative | Negative | SGA | 31.0 | Microcephaly |
| 05 | ND | ND | Negative | AGA | 31.0 | Microcephaly |
| 06 | **Positive** | Negative | Negative | AGA | 31.0 | Microcephaly |
| 07 | **Positive** | **Positive** | Negative | SGA | 31.0 | Microcephaly |
| 08 | Negative | Negative | **Positive** | SGA | 31.0 | Microcephaly |
| 09 | **Positive** | Negative | Negative | SGA | 31.5 | Microcephaly |
| 10 | **Positive** | **Positive** | **Positive** | AGA | 31.5 | Microcephaly |
| 11 | **Positive** | Negative | **Positive** | SGA | 29.5 | Microcephaly |
| 12 | ND | ND | Negative | SGA | 31.0 | Microcephaly |
| 13 | **Positive** | **Positive** | **Positive** | SGA | 32.0 | Microcephaly |
| 14 | **Positive** | Negative | Negative | AGA | 31.0 | Microcephaly |
| 15 | Negative | Negative | Negative | AGA | 29.5 | Microcephaly |
| 16 | Negative | Negative | **Positive** | SGA | 25.5 | Severe microcephaly |
| 17 | **Positive** | Negative | Negative | SGA | 31.5 | Microcephaly |
| 18 | ND | ND | Negative | SGA | 31.5 | Microcephaly |
| 19 | ND | ND | Negative | SGA | 32.0 | Microcephaly |
| 20 | **Positive** | Negative | **Positive** | AGA | 31.0 | Microcephaly |
| 21 | **Positive** | Negative | Negative | SGA | 31.0 | Microcephaly |
| 22 | **Positive** | Negative | Negative | SGA | 30.5 | Microcephaly |
| 23 | **Positive** | **Positive** | Negative | SGA | 30.0 | Microcephaly |
| 24 | **Positive** | Negative | Negative | SGA | 31.0 | Microcephaly |
| 25 | **Positive** | Negative | Negative | AGA | 29.5 | Severe microcephaly |

Abbreviations: AGA, appropriate for gestational age; SGA, small for gestational age; ND, not done.

**Supporting information S2.** Laboratory and clinical characteristics of 17 normocephalic newborns with congenital Zika infection (IgM anti-Zika virus or Zika virus RT-qPCR positive).

| Subject ID | IgG anti-Zika virus | IgM anti-Zika virus | Zika virus RT-qPCR | Size for gestational age | Head circumference, cm | Intergrowth classification |
| --- | --- | --- | --- | --- | --- | --- |
| 01 | **Positive** | Negative | **Positive** | AGA | 32.5 | Normocephaly |
| 02 | **Positive** | Negative | **Positive** | AGA | 33.0 | Normocephaly |
| 03 | **Positive** | Negative | **Positive** | SGA | 33.5 | Normocephaly |
| 04 | Negative | Negative | **Positive** | AGA | 35.0 | Normocephaly |
| 05 | Negative | Negative | **Positive** | AGA | 35.0 | Normocephaly |
| 06 | **Positive** | Negative | **Positive** | AGA | 34.0 | Normocephaly |
| 07 | **Positive** | Negative | **Positive** | AGA | 35.5 | Normocephaly |
| 08 | ND | ND | **Positive** | AGA | 32.5 | Normocephaly |
| 09 | **Positive** | Negative | **Positive** | AGA | 32.0 | Normocephaly |
| 10 | **Positive** | Negative | **Positive** | AGA | 34.0 | Normocephaly |
| 11 | **Positive** | Negative | **Positive** | SGA | 33.0 | Normocephaly |
| 12 | **Positive** | Negative | **Positive** | AGA | 36.0 | Normocephaly |
| 13 | **Positive** | ND | **Positive** | SGA | 33.0 | Normocephaly |
| 14 | **Positive** | **Positive** | Negative | AGA | 33.5 | Normocephaly |
| 15 | Negative | **Positive** | Negative | AGA | 29.0 | Normocephaly |
| 16 | **Positive** | **Positive** | Negative | AGA | 37.0 | Normocephaly |
| 17 | **Positive** | **Positive** | Negative | AGA | 35.5 | Normocephaly |

Abbreviations: AGA, appropriate for gestational age; SGA, small for gestational age; ND, not done.
